# Cellular senescence by loss of *Men1* in osteoblasts is critical for age‐related osteoporosis

**DOI:** 10.1111/acel.14254

**Published:** 2024-06-22

**Authors:** Yuichiro Ukon, Takashi Kaito, Hiromasa Hirai, Takayuki Kitahara, Masayuki Bun, Joe Kodama, Daisuke Tateiwa, Shinichi Nakagawa, Masato Ikuta, Takuya Furuichi, Yuya Kanie, Takahito Fujimori, Shota Takenaka, Tadashi Yamamuro, Satoru Otsuru, Seiji Okada, Masakatsu Yamashita, Takeshi Imamura

**Affiliations:** ^1^ Department of Orthopaedic Surgery Osaka University Graduate School of Medicine Suita Osaka Japan; ^2^ Department of Orthopedics University of Maryland School of Medicine Baltimore Maryland USA; ^3^ Department of Orthopaedic Surgery Osaka General Medical Center Osaka Osaka Japan; ^4^ Division of Endocrinology, Diabetes and Metabolism Beth Israel Deaconess Medical Center and Harvard Medical School Boston Massachusetts USA; ^5^ Department of Immunology Ehime University Graduate School of Medicine Toon Ehime Japan; ^6^ Department of Molecular Medicine for Pathogenesis Ehime University Graduate School of Medicine Toon Ehime Japan

**Keywords:** AMPK, cellular senescence, Men1, mTORC1, osteoporosis

## Abstract

Recent evidence suggests an association between age‐related osteoporosis and cellular senescence in the bone; however, the specific bone cells that play a critical role in age‐related osteoporosis and the mechanism remain unknown. Results revealed that age‐related osteoporosis is characterized by the loss of osteoblast *Men1*. Osteoblast‐specific inducible knockout of *Men1* caused structural changes in the mice bones, matching the phenotypes in patients with age‐related osteoporosis. Histomorphometrically, *Men1*‐knockout mice femurs decreased osteoblastic activity and increased osteoclastic activity, hallmarks of age‐related osteoporosis. Loss of *Men1* induces cellular senescence via mTORC1 activation and AMPK suppression, rescued by metformin treatment. In bone morphogenetic protein‐indued bone model, loss of *Men1* leads to accumulation of senescent cells and osteoporotic bone formation, which are ameliorated by metformin. Our results indicate that cellular senescence in osteoblasts plays a critical role in age‐related osteoporosis and that osteoblast‐specific inducible *Men1*‐knockout mice offer a promising model for developing therapeutics for age‐related osteoporosis.

## Graphical abstract

Osteoblast‐specific inducible Men1 knockout mimics age‐related osteoporosis even in young mice. Men1 deletion in osteoblasts promotes replicative stress‐induced cellular senescence presumably through mTORC1 activation and AMPK suppression, rescued by metformin treatment.

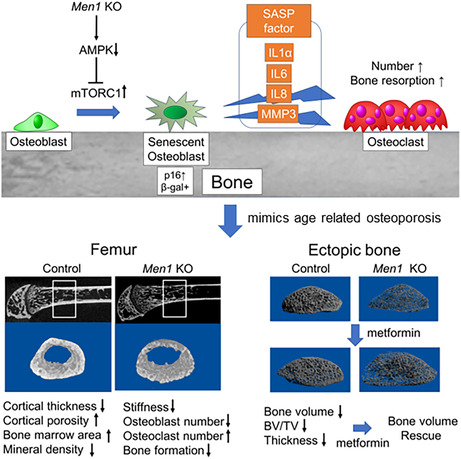

**Abbreviations**
BFR/BS: bone formation rate per bone surface
BMP: bone morphogenic protein
BV/TV: bone volume/tissue volume
MAR: mineral apposition rate
MEN1: multiple endocrine neoplasia type 1
N.Ob/BS: number of osteoblasts per bone surface
N.Oc/BS: number of osteoclasts per bone surface
Ob.S/BS: osteoblast surface per bone surface
Oc.S/BS: osteoclast surface per bone surface
OIS: oncogene‐induced senescence
RANKL: receptor activator of NF‐κB ligand
RT‐qPCR: reverse transcription‐quantitative polymerase chain reaction
SASP: senescence‐associated secretory phenotype
SA‐β Gal: senescence‐associated β‐galactosidase
TAM: tamoxifen
Tb.Th: trabecular thickness
TGF‐β: transforming growth factor‐beta
α‐MEM: alpha‐minimal essential medium

## 1 INTRODUCTION

Osteoporosis is a systemic skeletal disease characterized by low bone mass and the deterioration of bone microarchitecture, which increase the occurrence of fragility fractures (Aspray & Hill, 2019; Ukon et al., 2019). Among the various factors affecting osteoporosis, aging, menopause, and decreased activity, age‐related osteoporosis is becoming important because an increase in aging population, which started in high‐income countries is now occurring even in low‐ and middle‐income countries (Gregson et al., 2022).

Accumulating evidence highlights a close relationship between age‐related diseases and cellular senescence (Baker et al., 2016), which was discovered by Hayflick et al. (Hayflick & Moorhead, 1961) and is defined by an irreversible cell growth arrest regulated through the p16^ink4a^/RB and p53/p21^CIP1^ pathways (Muñoz‐Espín & Serrano, 2014). Senescent cells acquire a pro‐inflammatory phenotype, known as the senescence‐associated secretory phenotype (SASP), which leads to the disruption of tissue and organ functions and is considered to be a major cause of various chronic diseases (Regulski, 2017). Therefore, treatment strategies for chronic diseases targeting the removal of senescent cells (senolytics) or inhibition of the SASP (senomorphics) have recently come into the research spotlight (Kirkland & Tchkonia, 2020).

The accumulation of senescent cells with age has also been confirmed to occur in the bones, suggesting cellular senescence as a key regulator of age‐related osteoporosis (Farr et al., 2016); thus, it is rational to develop treatment strategies for osteoporosis that target senescent cells (Farr & Khosla, 2019). Indeed, inhibition of the negative effects of senescent cells in mice attenuated the rate of bone loss with age in mice (Farr et al., 2017). However, the precise mechanism by which bone cells become senescent during the natural aging process remains largely unknown. Understanding this mechanism will enable the development of more precise therapies for age‐related osteoporosis.

*Men1* gene is originally known to be a tumor suppressor for multiple endocrine neoplasia type 1 (MEN1) syndrome (Thakker et al., 2012). We previously found *Men1* deficiency in T cells induces cellular senescence‐associated immunodeficiency (Kuwahara et al., 2014; Suzuki et al., 2018) and focused on a possible role of *Men1* gene in osteoblast senescence. Our current results indicate loss of *Men1* is a critical driver for osteoblast senescence, and osteoblast‐specific inducible *Men1* knockout mice could be the first animal model for age‐related osteoporosis.

## 2 RESULTS

### 2.1 *Men1* mRNA levels are significantly reduced in the osteoporotic bones of aged mice

**FIGURE 1:**
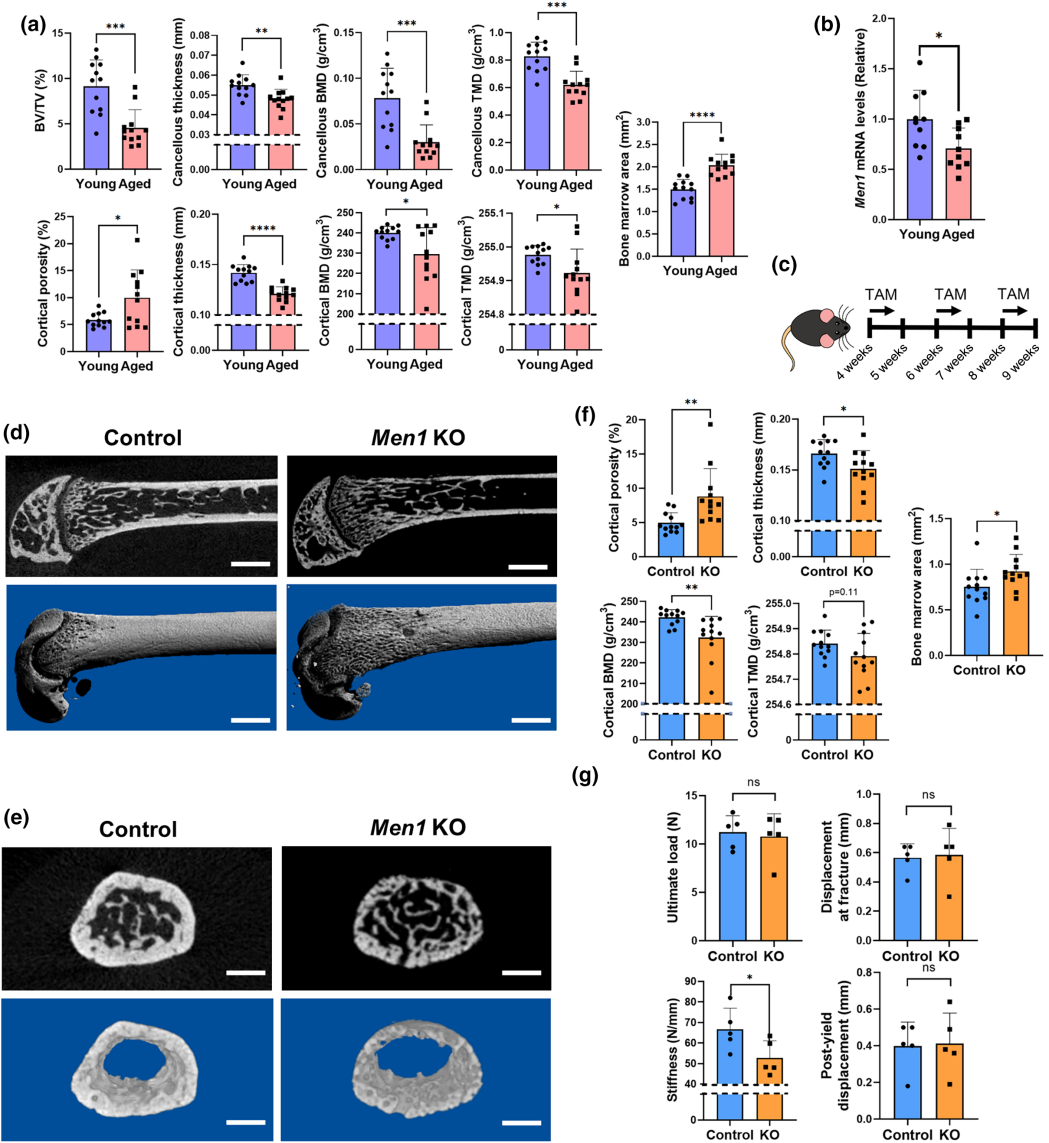
Structural analysis of bones in the trunk. (a) Micro‐computed tomography (μCT) analysis of the femur of wild‐type 2‐month‐old (young, *n* = 12) and 24‐month‐old (aged, *n* = 12) mice, including bone volume (BV)/tissue volume (TV), cancellous thickness, cancellous bone mineral density (BMD), cancellous tissue mineral density (TMD), cortical porosity, cortical thickness, cortical BMD, cortical TMD, and bone marrow area. (b) Relative *Men1* mRNA levels to young mice in the spine bone. Young mice (*n* = 10), aged mice (*n* = 10). (c) Experimental design for deleting the *Men1* gene of osteoblasts. *Men1* ^flox/flox^ mice (Control) and *Men1* ^flox/flox^; *Col1a1‐cre/ERT2* mice (*Men1* KO) received tamoxifen (TAM) treatment at 10 mg/kg/day for 4 days at 4, 6, and 8 weeks of age. Mice were euthanized and analyzed at 9 weeks of age. (d) Representative μCT sagittal images of the Control and *Men1* KO femur from 9‐week‐old mice. Upper: two‐dimensional image; lower: three‐dimensional reconstructed image. Scale bars, 1 mm. (e) Representative μCT axial images of the Control and *Men1* KO femur from 9‐week‐old‐ mice at 1.5 mm proximal to the distal growth plate. Upper: two‐dimensional image; lower: three‐dimensional cortical bone image. Scale bars, 1 mm. (f) Micro‐CT analysis of the femur of 9‐week‐old Control (*n* = 12) and *Men1* KO (*n* = 12) mice, including cortical porosity, cortical thickness, cortical BMD, cortical TMD, and bone marrow area. (g) Result of biomechanical testing in 9‐week‐old Control (*n* = 5) and *Men1* KO (*n* = 5) mice, including ultimate load, displacement at fracture, stiffness, and post‐yield displacement. Data represent mean ± SD (error bars). *p < 0.05, **p < 0.01, ***p < 0.001, ****p < 0.0001 by two‐tailed Student's *t*‐test (unpaired). ns, not statistically significant.

Recent studies suggest that cellular senescence is a key regulator of age‐related osteoporosis (Farr et al., 2016). We previously identified the tumor suppressor gene *Men1* as a regulator of cellular senescence regulator in T cells (Kuwahara et al., 2014; Suzuki et al., 2018). These findings prompted us to examine whether *Men1* also played a critical role in osteoporosis during aging. To test this hypothesis, we initially compared femurs of 2‐month‐old (young mice) and 24‐month‐old mice (aged mice) using microcomputed tomography (μCT). Evaluation of the femurs of young and aged mice via μCT showed a decrease in the bone volume (BV)/total volume (TV) and the cancellous and cortical bone thickness, bone mineral density (BMD), tissue mineral density (TMD), and an increase in the bone marrow area in 24‐month‐old mice compared with those in 2‐month‐old mice (Figure 1a). The bone phenotypes in 24‐month‐old mice resembled those of older humans with osteoporosis. Moreover, the mRNA levels of *Men1* were reduced in 24‐month‐old mice spine bone (Figure 1b), accompanied by increased mRNA levels of cellular senescence genes (*p16*, *p21*, *p53*, *Il1α*, *Il8*, and *MMP3*) compared with those of 2‐month‐old mice spine bone (Figure S1). For the purpose of overall aging assessment, spine bone were used in Figure 1b; Figure S1 as previously reported (Farr et al., 2016). These results suggest that the loss of *Men1* is closely involved in the pathology of age‐related osteoporosis.

### 2.2 *Men1* deletion in osteoblasts induces osteoporotic changes on cortical bone even in young mice

To further identify the role of *Men1* on age‐related osteoporosis, we generated *Men1* ^flox/flox^; *Col1a1‐cre/ERT2* mice that can delete osteoblast *Men1* by tamoxifen (TAM) treatment. We used inducible osteoblast‐specific mice because cellular senescence is deeply involved during the embryonic period (Muñoz‐Espín et al., 2013), thus developmental *Men1* effect cannot be eliminated by permanent osteoblast‐specific *Men1* KO mice. Femur bone of 9‐week‐old *Men1* ^flox/flox^; *Col1a1‐Cre/ERT2* mice treated with TAM (4 days/week starting at 4, 6, and 8 weeks of age; Figure 1c) were evaluated by μCT. The μCT images clearly demonstrated thinning of the cortical bone in the distal femur of TAM treated *Men1* ^flox/flox^; *Col1a1‐cre/ERT2* (*Men1* KO) mice compared to *Men1* ^flox/flox^ (Control) mice (Figure 1d,e). Microstructural analyses showed that the cortical bone in the *Men1* KO mice was thinner, more porotic, and had lower mineral density, with a wider bone marrow area than that in Control mice (Figure 1f). These results suggest that *Men1* deletion in osteoblasts predominantly affects the osteoporotic phenotype in the cortical bone. Since the cortical bone microstructure has a great influence on bone strength (Bala et al., 2015), we investigated the bone strength of *Men1* KO mice by a mechanical testing. The ultimate load, displacement at fracture, and post‐yield displacement were comparable between Control and *MenEN1* KO mice (Figure 1g); however, the femoral stiffness was decreased in *Men1* KO mice (Figure 1g). Thus, femurs from *Men1* KO mice resembled the fragile bones of older humans in terms of both bone structure and mechanical strength.

### 2.3 *Men1* deletion induces osteoblast senescence, attenuates osteoblastic activity, and increases osteoclastic activity in vivo

**FIGURE 2:**
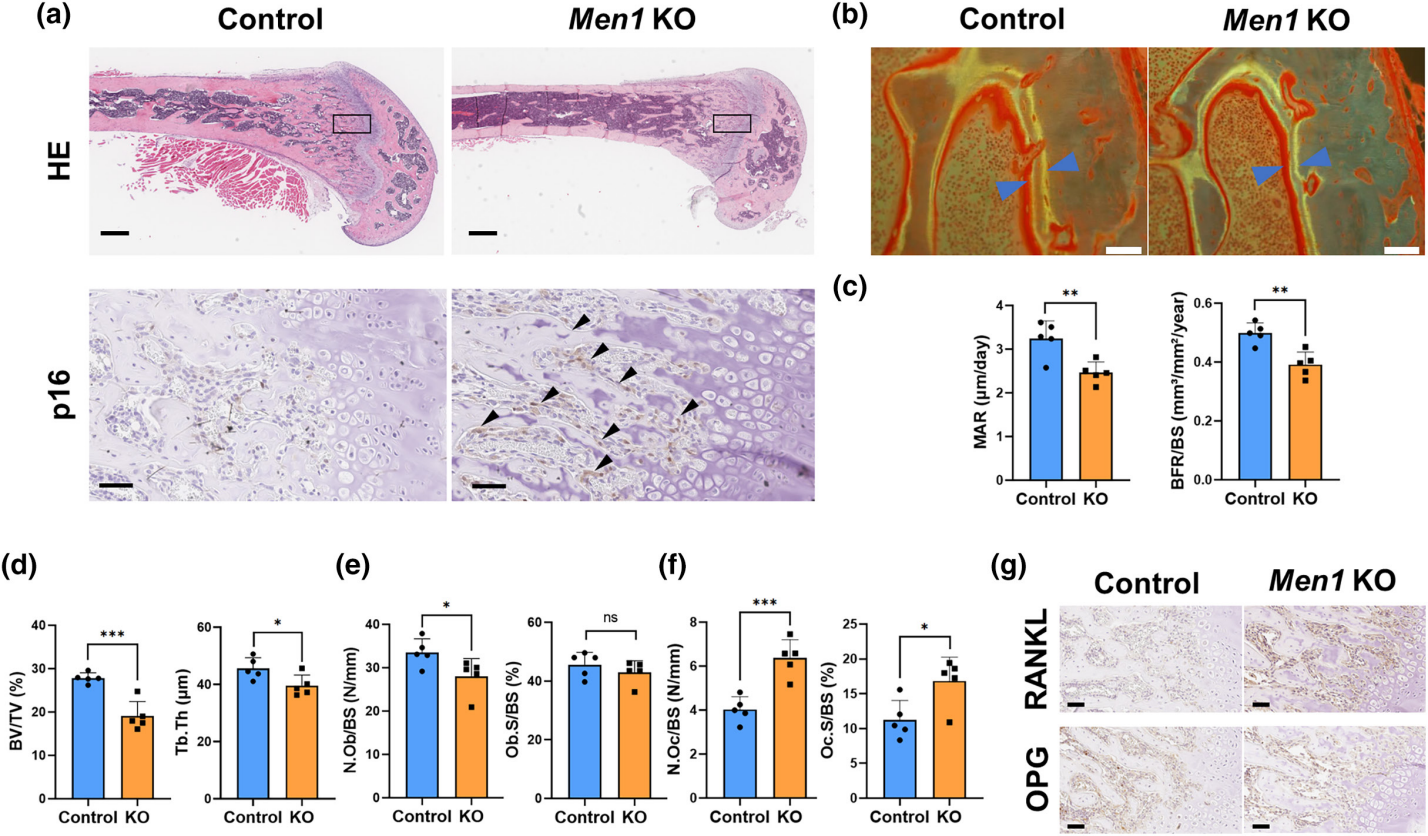
Histological characteristics of *Men1‐*deficient bones. (a) Representative histological images of the femur bone from 9‐week‐old *Men1* ^flox/flox^ (Control) mice and *Men1* ^flox/flox^; *Col1a1‐cre/ERT2* (*Men1* KO) mice. Hematoxylin and eosin staining (HE) and p16 immunostaining. Lower panels are enlarged images of the rectangular area of the upper image. Arrows indicate p16‐positive cells. Scale bars, 500 μm (upper) and 50 μm (lower). (b) Representative fluorescent images of the femur from 9‐week‐old Control and *Men1* KO mice. Scale bars, 50 μm. Arrows indicate the space between the double‐stained lines. (c) Histomorphometric parameters of bone formation in 9‐week‐old Control (*n* = 5) and *Men1* KO (*n* = 5) mice, including mineral apposition rate (MAR) and bone formation rate (BFR)/bone surface (BS). (d) Histomorphometric parameters of bone structure in 9‐week‐old Control (*n* = 5) and *Men1* KO (*n* = 5) mice, including bone volume (BV)/tissue volume (TV) ratio and trabecular thickness (Tb.Th). (e) Histomorphometric parameters of osteoblasts in 9‐week‐old Control (*n* = 5) and *Men1* KO (*n* = 5) mice, including number of osteoblasts (N.Ob)/BS and osteoblast surface (Ob.S)/BS. (f) Histomorphometric parameters of osteoclasts in 9‐week‐old Control (*n* = 5) and *Men1* KO (*n* = 5) mice, including number of osteoclasts (N.Oc)/BS and osteoclast surface (Oc.S)/BS. (g) Representative RANKL, OPG immunostaining images of the femur bone from 9‐week‐old Control and *Men1* KO mice. Scale bars, 50 μm. Data represent mean ± SD (error bars). *p < 0.05, **p < 0.01, ***p < 0.001 by two‐tailed Student's *t*‐test (unpaired). ns, not statistically significant.

After identifying the structural phenotype, we next investigated the detailed actions of *Men1* deficiency histologically. Cortical thinning due to *Men1* deletion was observed, similar to the μCT findings (Figure 2a). Furthermore, *Men1* KO mice exhibited an increase in the number of p16‐positive osteoblasts (Figure 2a), which was consistent with the area where *Cre/loxP* recombination was confirmed (Figure S3a). These results suggest that *Men1* deficiency in vivo leads to osteoblast senescence. To elucidate the effect of *Men1* deletion in osteoblasts on bone formation, bone histomorphometry analysis was performed using the femurs of Control and *Men*1 KO mice. The spaces between the double‐stained lines were narrow (Figure 2b), which matched the decrease in osteogenic ability (as assessed by the mineral apposition rate [MAR] and bone formation rate per bone surface ratio [BFR/BS]) in *Men1* KO mice (Figure 2c). *Men1* KO mice exhibited decreased bone area and thickness, reflecting their low osteogenic ability (Figure 2d), along with a decrease in the number of osteoblasts and the area of the osteoblast surface (Figure 2e). The osteoblast‐related bone formation indices were all generally decreased in *Men1* KO mice, matching the general findings in aged humans. Under normal conditions, the number of osteoclasts generally parallels the number of osteoblasts, regulated by cross‐talk mediated through the receptor activator of NF‐κB ligand (RANKL) and RANK (Yasuda, 2021). Interestingly, histomorphometry analysis of bone resorption parameters showed an increase in the number of osteoclasts and the area of the osteoclast surface in *Men1* KO mice (Figure 2f), although the number of osteoblasts decreased. Immunostaining for RANKL/OPG revealed extensive RANKL upregulation in *Men1* KO, while OPG did not differ compared to those in Control mice (Figure 2g). This suggests that the mechanism of osteoclast increase in *Men1* KO mice may involve an elevated RANKL/OPG ratio. Thus, *Men1* deficiency widened the gap between bone formation and resorption, a characteristic of age‐related osteoporosis (Ukon et al., 2019).

### 2.4 *Men1* deletion in osteoblasts promotes replicative stress‐induced cellular senescence through presumably mTORC1

**FIGURE 3:**
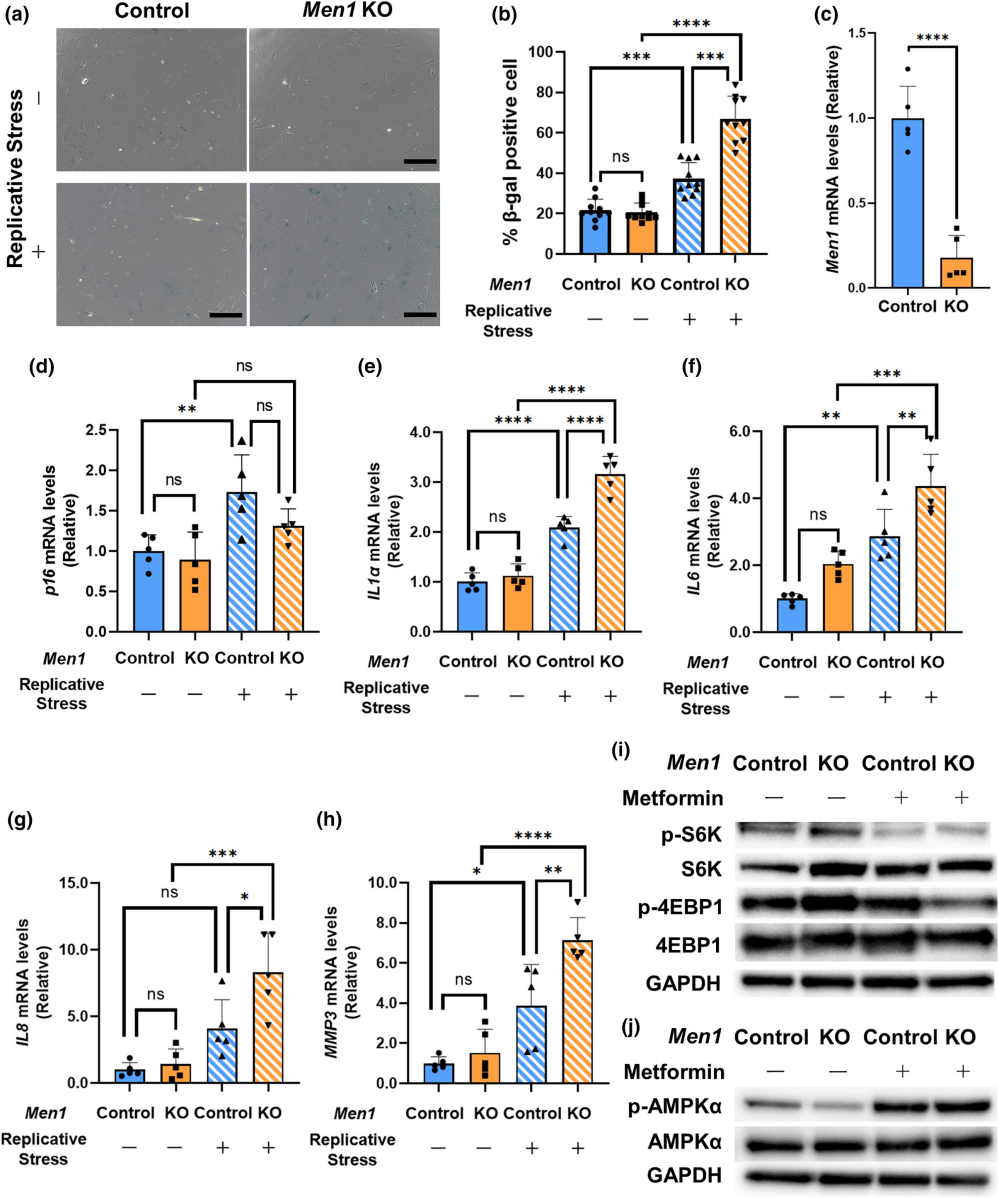
Osteoblast senescence induced by *Men1* deficiency in vitro. (a) Senescence‐associated galactosidase (SA β‐Gal) staining of osteoblasts from the *Men1* ^flox/flox^ murine mice calvaria. Osteoblasts were infected with eGFP adenovirus (Ad‐GFP, Control) and Cre recombinase adenovirus (Ad‐Cre‐GFP, *Men1* KO). Passage (P) 3 (replicative stress: −) and P6 (Replicative stress: +) cells were used for the experiments. Scale bars, 250 μm. (b) Quantitative evaluation of β‐Gal‐positive cells in Control (*n* = 10 fields) and *Men1* KO (*n* = 10 fields) mice. (c) Relative mRNA levels of *Men1* to Control osteoblasts (Replicative stress: −). Control osteoblasts (Replicative stress: −), *n* = 5; *Men1* KO osteoblasts (Replicative stress: −), *n* = 5. (d–h) Relative mRNA levels of *p16*, *IL1α*, *IL6*, *IL8*, and *MMP3* to Control osteoblasts (Replicative stress: −). Control osteoblasts (Replicative stress: −) (*n* = 5), *Men1* KO osteoblasts (Replicative stress: −) (*n* = 5), Control osteoblasts (Replicative stress: +) (*n* = 5), and *Men1* KO osteoblasts (Replicative stress: +) (*n* = 5). (i) Immunoblotting to evaluate the expression of factors downstream of mTORC1. (j) Immunoblotting of AMPKα in control osteoblasts (Replicative stress: −), *Men1* KO osteoblasts (Replicative stress: −), Control osteoblasts (Replicative stress: +), and *Men1* KO osteoblasts (Replicative stress: +). Data represent mean ± SD (error bars). *p < 0.05, **p < 0.01, ***p < 0.001, ****p < 0.0001 by two‐tailed Student's *t*‐test (unpaired) (c), one‐way ANOVA followed by Sidak's test (b, d–h). ns, not statistically significant.

The results summarized above demonstrate that *Men1* deletion in osteoblasts resulted in structural changes in the cortical bone (Figure 1f) and induced p16 expression in the metaphysis (Figure 2a). However, the mechanistic link between *Men1* deletion in osteoblasts and age‐related osteoporosis remained unclear. Since *Men1* was previously shown to act as a cellular senescence regulator in T cells (Kuwahara et al., 2014; Suzuki et al., 2018), we next evaluated whether *Men1* deficiency directly causes cellular senescence using in vitro adenoviral‐mediated *Men1* KO in primary osteoblasts from *Men1* ^flox/flox^ mice. Osteoblasts were infected with an adenovirus expressing green fluorescent protein (Ad‐GFP; Control) or Cre (Ad‐Cre‐GFP; *Men1* KO) and cellular senescence was induced by replicative stress, as described in the Materials and Methods section. After multiple passages, the positivity of senescence‐associated β‐galactosidase (SA‐β Gal) staining was significantly increased in *Men1* KO osteoblasts compared with that in the control osteoblasts (Figure 3a,b). To further verify the characteristics of each osteoblast type, the mRNA levels of cellular senescence‐associated genes and *Men1* were investigated. *Cre/loxP* recombination was confirmed by *Men1* reduction (Figure 3c). The induction of cellular senescence induced by replicative stress was confirmed by higher mRNA levels of *p16* (Figure 3d) in both Control and *Men1* KO osteoblasts. Even under the same condition of replicative stress, the mRNA levels of SASP genes (*Il1α*, *IL6*, *IL8*, and *MMP3*) were increased in *Men1* KO compared to those in Control osteoblasts (Figure 3e–h). Since SASP levels are elevated during the late stages of cellular senescence (Muñoz‐Espín & Serrano, 2014), these results suggest that the loss of *Men1* in osteoblasts accelerates cellular senescence induced by replicative stress. To elucidate the mechanism underlying replicative stress‐induced senescence in osteoblasts with *Men1* deletion, we investigated the potential involvement of the mTORC1 pathway, one of the major signaling pathways for cellular senescence (Weichhart, 2018). Phosphorylation of S6K and 4EBP1, which are downstream effectors of mTORC1, was enhanced by *Men1* deletion, and metformin treatment suppressed activation of the mTORC1 pathway caused by *Men1* deficiency (Figure 3i). The quantification data also showed the same mTORC1 changes (Figure S2a). We performed additional immunoblotting and its quantification to understand the mechanism by which mTORC1 was suppressed by metformin. The findings showed that phosphorylation of AMPKα, which suppresses mTORC1 (Jeon, 2016), was significantly reduced by *Men1* deficiency and restored by metformin treatment (Figure 3j; Figure S2b). However, no clear involvement of Akt, ERK1/2, and GSK3β was observed (Figures S2c,d). These findings suggest that replicative stress‐induced senescence in osteoblasts with Men1 deletion is closely associated with mTORC1 pathway activation, which can be restored through AMPK activation by metformin treatment.

### 2.5 Cellular senescence and a decrease in *Men1* are closely involved in bone formation during natural aging

**FIGURE 4:**
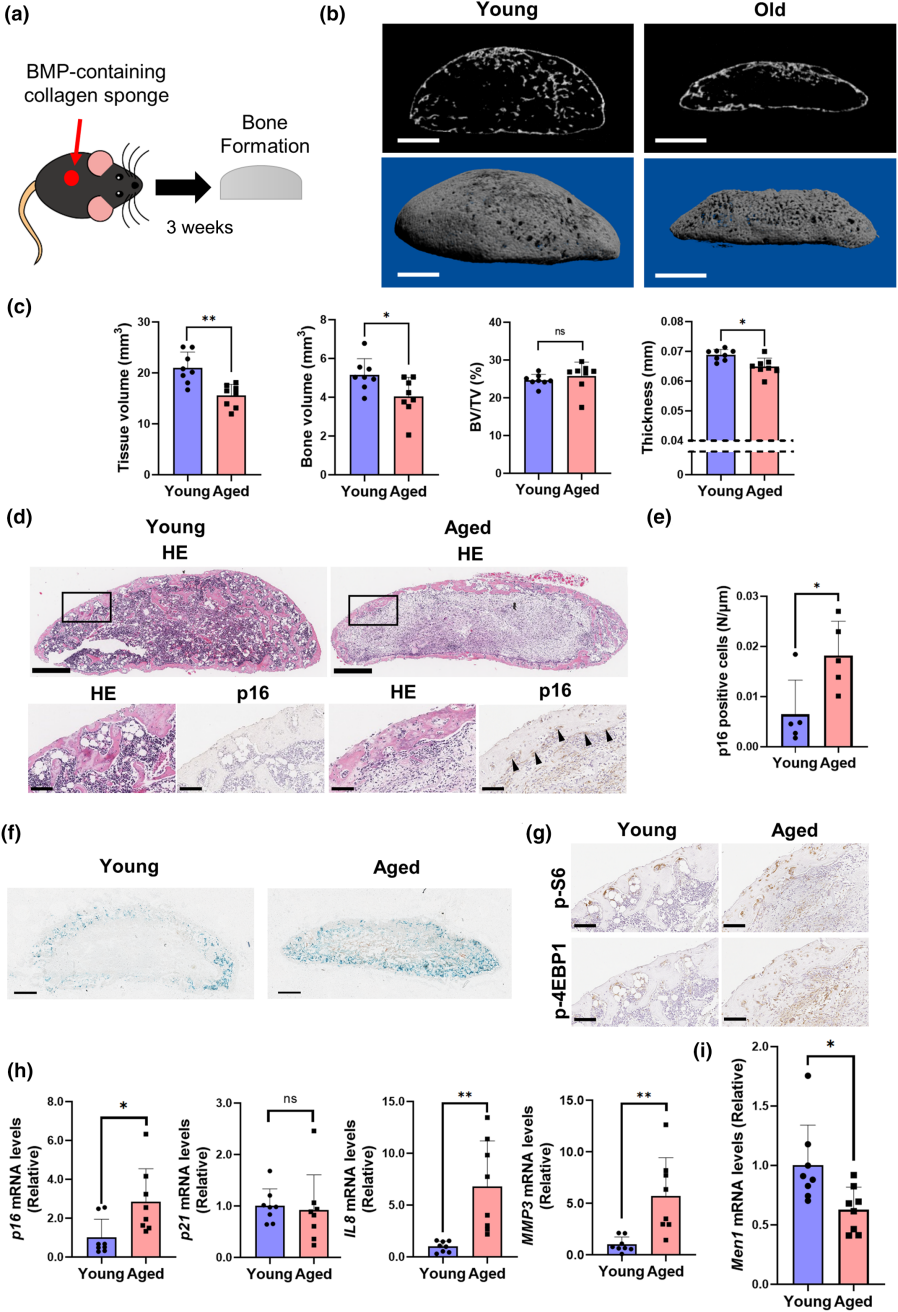
Senescent bone formation in natural aging. (a) Schema of establishment of the ectopic bone formation model. A BMP‐containing collagen sponge was set underneath the fascia of 2‐month‐old (young) and 24‐month‐old mice (aged) mice. After 3 weeks, the formed ectopic bone was evaluated. (b) Representative μCT sagittal images of the ectopic bone. Scale bars, 1 mm. (c) Micro‐CT analysis of the ectopic bone in young (*n* = 8) and aged (*n* = 8) mice, including tissue volume (TV), bone volume (BV), BV/TV, and thickness. (d) Representative histological images of the ectopic bone in young and aged mice. Hematoxylin and eosin staining (HE) and p16 immunostaining. Lower panels are enlarged images of the rectangular area of upper panels. Arrows indicate p16‐positive cells. Scale bars, 500 μm (upper) and 100 μm (lower). (e) Quantitative evaluation of p16‐positive cells in young (*n* = 5) and aged (*n* = 5) mice. (f) Representative beta‐galactosidase staining images of the ectopic bone in young and aged mice. Scale bars, 500 μm. (g) Representative histological images of the ectopic bone in young and aged mice. p‐S6 and p‐4EBP1 immunostaining. Scale bars, 100 μm. (h) Relative mRNA levels of *p16*, *p21*, *IL1α*, *IL8*, and *MMP3* to young mice. Young mice (*n* = 8) and aged mice (*n* = 8). (i) Relative *Men1* mRNA levels to young mice. Young mice (*n* = 8) and aged mice (*n* = 8). *p < 0.05, **p < 0.01 by two‐tailed Student's *t*‐test (unpaired). Data represent mean ± SD (error bars). ns, not statistically significant.

Femoral analysis of *Men1* KO mice represented signs of cellular senescence, and osteoblast senescence presumably via mTORC1 induced by *Men1* deficiency was confirmed in vitro. To further clarify the relevance of cellular senescence in vivo due to *Men1* deficiency, we established a model of accelerated cellular senescence in bone tissue. Bone morphogenic protein (BMP) is known to activate mTORC1 and regulate cellular senescence (Kaneda et al., 2011; Karner et al., 2017). A previous report showed that the transplantation of a BMP‐containing collagen sponge allowed us to observe the bone formation capacity (Hashimoto et al., 2020). We hypothesized that the effects of osteoblast senescence would be enhanced by conditions of BMP‐induced bone formation. Experiments were conducted using BMP‐containing collagen sponges (Figure 4a). BMP‐induced ectopic bones in 24‐month‐old mice were significantly thinner and smaller than those in 2‐month‐old mice (Figure 4b,c), as observed during human aging (Goodnough & Goodman, 2022). We further investigated cellular senescence in the ectopic bone using histological evaluation and reverse transcription‐quantitative polymerase chain reaction (RT‐qPCR). Immunostaining revealed that the number of p16‐positive cells was significantly increased on the surface of the ectopic bone from aged mice (Figure 4d,e). The areas positively stained for β‐Gal were also increased in the aged mice (Figure 4f). In addition, an increase in p‐4EBP1 and p‐S6 was observed in aged mice, suggesting activation of mTORC1 (Figure 4g). The mRNA levels of *p16* and SASP factors increased in the ectopic bones of aged mice (Figure 4h). Additionally, similar to the results from the vertebral bones (Figure 1b; Figure S1), the mRNA levels of *Men1* decreased in the ectopic bones of aged mice (Figure 4i). Thus, the BMP‐induced ectopic bone model suggests that cellular senescence and a decrease in *Men1* mRNA levels are closely associated with low bone formation activity in aging individuals.

### 2.6 Metformin suppresses cellular senescence in ectopic bones from *Men1* KO mice

**FIGURE 5:**
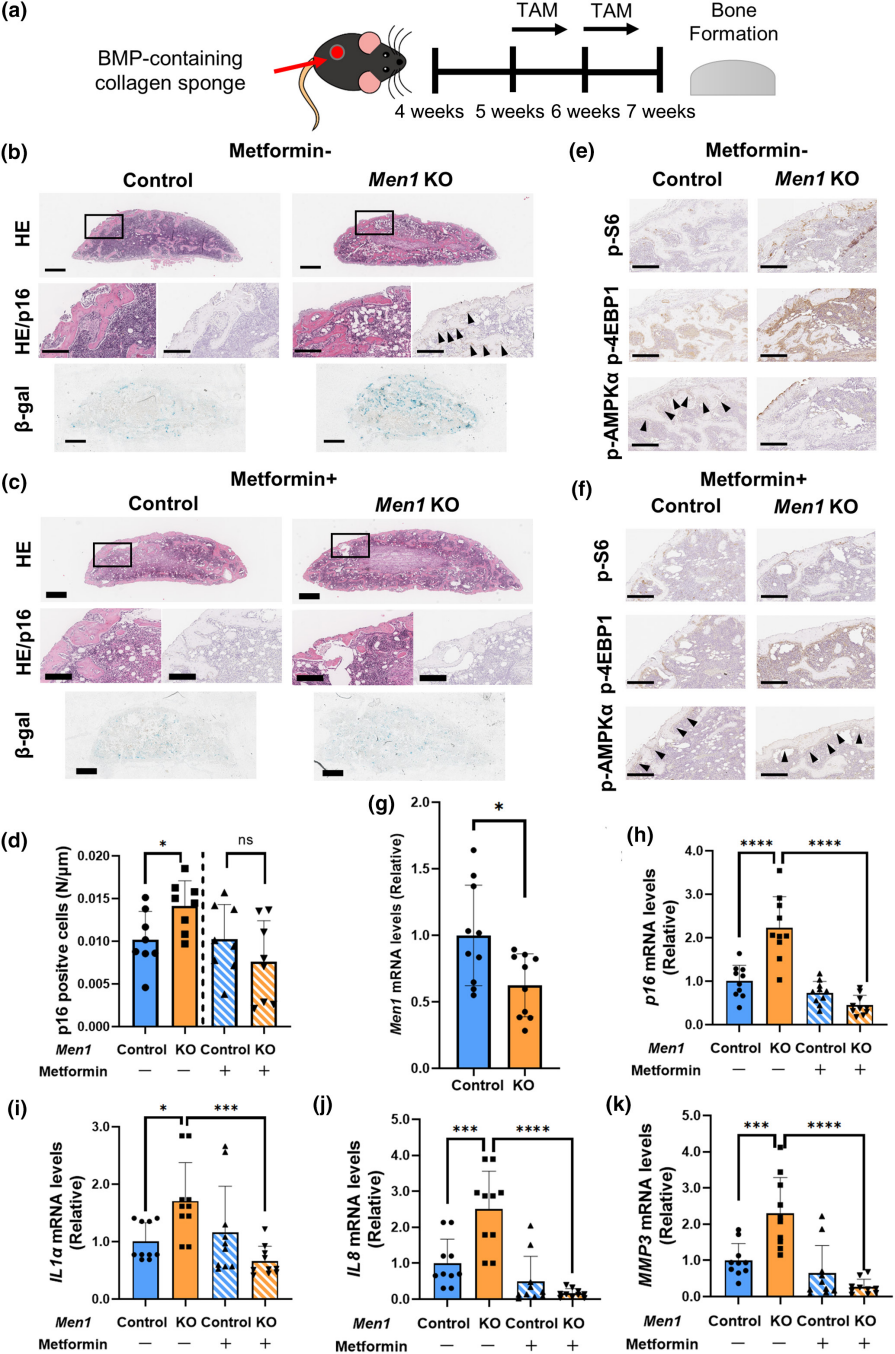
Characteristics of *Men1* knockout bone formation. (a) Schema of the ectopic bone formation model of *Men1* ^flox/flox^ (Control) and *Men1* ^flox/flox^; *Col1a1‐cre/ERT2* (*Men1* KO) mice. (b) Representative histological images of Control and *Men1* KO ectopic bone without metformin administration from 7‐week‐old mice. Hematoxylin and eosin staining (HE), p16 immunostaining, and beta‐galactosidase (β‐Gal) staining. Second (HE, p16) images are enlarged images of the rectangular area of the first (HE) images. Arrows indicate p16‐positive cells. Scale bars, 500 μm (first, third images) and 200 μm (second images). (c) Representative histological images of Control and *Men1* KO ectopic bone with metformin administration from 7‐week‐old mice. HE, p16 immunostaining, and β‐Gal staining. Second (HE, p16) images are enlarged images of the rectangular area of the first (HE) images. Scale bars, 500 μm (first, third images) and 200 μm (second images). (d) Quantitative evaluation of p16‐positive cells relative to Control bone (metformin: −) (*n* = 8) in Control bone (metformin: −) (*n* = 8), *Men1* KO bone (metformin: −) (*n* = 8), Control bone (metformin: +) (*n* = 8), and *Men1* KO bone (metformin: +) (*n* = 8) from 7‐week‐old mice. (e) Representative immunostaining images for p‐S6, p‐4EBP1, and p‐AMPKα of Control and *Men1* KO ectopic bone without metformin administration from 7‐week‐old mice. Arrows indicate p‐AMPKα positive cells. Scale bars, 200 μm. (f) Representative immunostaining images for p‐S6, p‐4EBP1, and p‐AMPKα of Control and *Men1* KO ectopic bone with metformin administration from 7‐week‐old mice. Arrows indicate p‐AMPKα positive cells. Scale bars, 200 μm. (g) Relative mRNA levels of *Men1* to Control bone (metformin: −) in Control bone (metformin: −) (*n* = 10), *Men1* KO bone (metformin: −) (*n* = 10) from 7‐week‐old mice. (h–k) Relative mRNA levels of *p16*, *IL1α*, *IL8*, and *MMP3* to Control bone (metformin: −) in Control bone (metformin: −) (*n* = 10), *Men1* KO bone (metformin: −) (*n* = 10), Control bone (metformin: +) (*n* = 10), and *Men1* KO bone (metformin: +) (*n* = 10) from 7‐week‐old mice. Data represent mean ± SD (error bars). *p < 0.05, ***p < 0.001, ****p < 0.0001 by two‐tailed Student's *t*‐test (unpaired) (d, g), one‐way ANOVA followed by Sidak's test (h–k). ns, not statistically significant.

Since metformin suppressed the mTORC1 pathway in *Men1* KO osteoblasts (Figure 3i), we sought to determine whether metformin could also reverse the cellular senescence due to the loss of *Men1*. Using the BMP‐induced ectopic bone model (Figure 5a), we found that *Men1* deletion in osteoblasts resulted in an increase in the number of p16‐positive cells and the β‐Gal‐positive areas (Figure 5b), which were markedly reduced after the administration of metformin (Figure 5c,d). Next, we performed immunostaining of p‐S6, p‐4EBP1, and p‐AMPKα to examine the involvement of mTORC1 activation. Without metformin treatment, *Men1* KO showed increased p‐S6 and p‐4EBP1 and decreased p‐AMPKα compared to Control mice (Figure 5e). However, metformin treatment decreased p‐S6 and p‐4EBP1 and increased p‐AMPKα in both Control and *Men1* KO mice compared to those observed in respective metformin untreated mice (Figure 5f). Consistent with in vitro findings, these findings suggest that metformin suppressed mTORC1 activation through AMPK due to *Men1* deficiency in vivo. RT‐qPCR on BMP‐induced ectopic bones confirmed that the mRNA levels of *Men1* were decreased in *Men1* KO mice (Figure 5g). No significant difference was observed in the mRNA levels of *Runx2*, *Sp7*, and *Alpl*, which are downstream of SMADs (Figure S4a). Similar to the results in the femur, *Rankl* increased in *Men1* KO while *Opg* showed no difference (Figure S4b). Consistent with the histological results, the mRNA levels of the SASP genes *p16*, *IL1α*, *IL8*, and *MMP3* were significantly increased in the ectopic bones from *Men1* KO mice, and this increase was suppressed following metformin administration (Figure 5h–k). These results indicate that *Men1* deficiency promotes cellular senescence, which could be reversed by metformin treatment.

### 2.7 Metformin partially restores the bone morphometry of BMP‐induced ectopic bone in *Men1* KO mice

**FIGURE 6:**
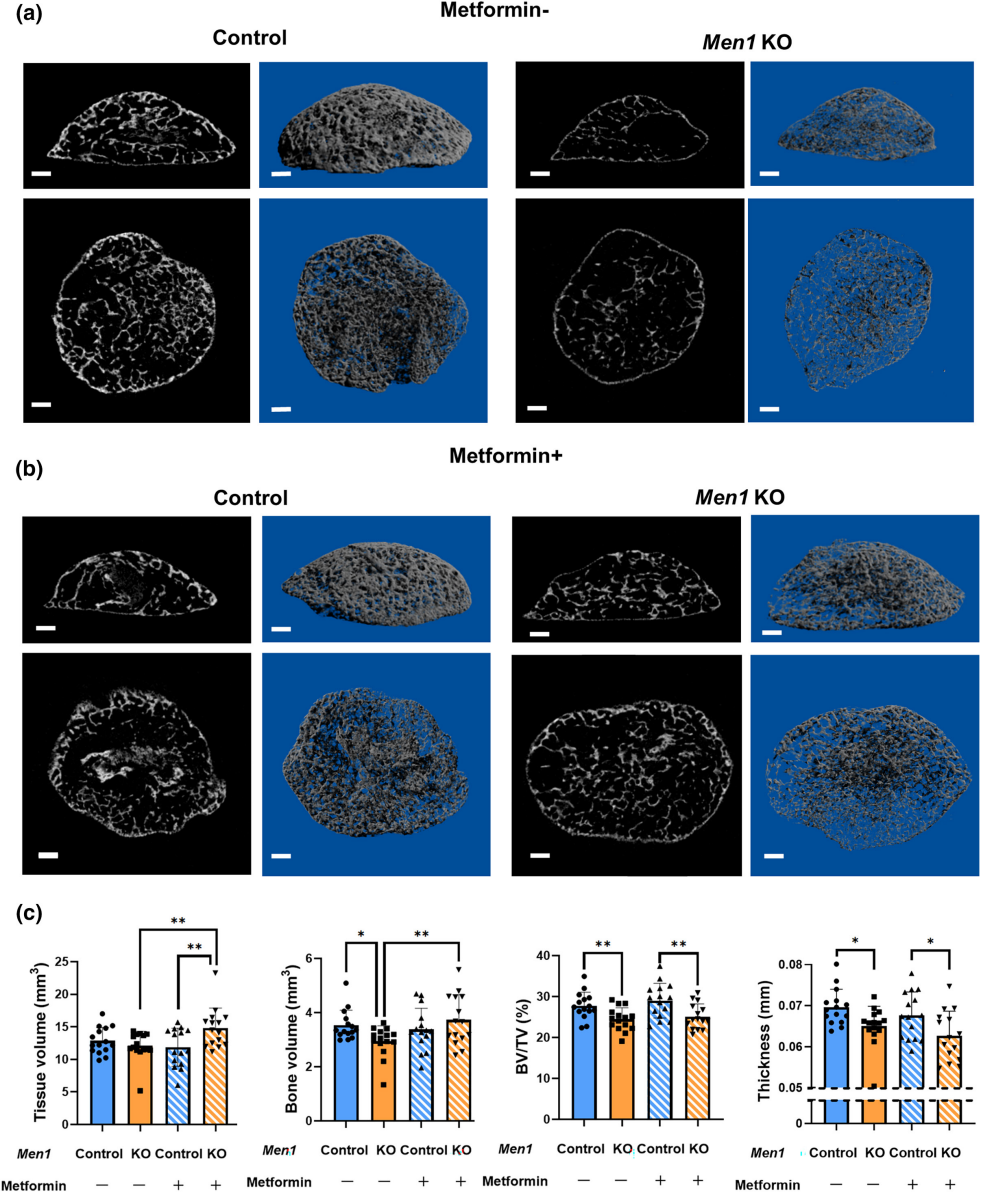
Miro‐computed tomography (μCT) analysis of ectopic bone with osteoblast senescence. (a) Representative μCT images of the ectopic bone of *Men1* ^flox/flox^ (Control) and *Men1* ^flox/flox^; *Col1a1‐cre/ERT2* (*Men1* KO) mice without metformin administration from 7‐week‐old mice. Sagittal (upper) and coronal (lower) images. Two‐dimensional images (left) and three‐dimensional reconstructed images (right). Scale bars, 500 μm. (b) Representative μCT images of the ectopic bone of Control and *Men1* KO mice with metformin administration from 7‐week‐old mice. Sagittal (upper) and coronal (lower) images. Two‐dimensional images (left) and three‐dimensional reconstructed images (right). Scale bars, 500 μm. (c) Micro‐CT analysis of the ectopic bone in Control (metformin: −) (*n* = 15), *Men1* KO (metformin: −) (*n* = 15), Control bone (metformin: +) (*n* = 15), and *Men1* KO (metformin: +) (*n* = 15) mice from 7‐week‐old mice, including tissue volume (TV), bone volume (BV), BV/TV, and thickness. Data represent mean ± SD (error bars). *p < 0.05, **p < 0.01 by one‐way ANOVA followed by Fisher's LDS test.

Both in vitro and in vivo, *Men1* deletion induced cellular senescence in osteoblasts, leading to reduced osteogenesis. Since cellular senescence in *Men1* KO mice was restored by metformin treatment, we examined whether metformin could also reverse the reduction in osteogenesis in *Men1* KO mice. The μCT images and three‐dimensional reconstruction showed bone thinning and a decrease in BV, BV/TV in the ectopic bone of the *Men1* KO mice (Figure 6a,c). Notably, metformin administration significantly increased the volume, but not the thickness, of the ectopic bones from *Men1* KO mice (Figure 6b,c). Taken together, our current results suggest that metformin partially restores osteogenesis reduced by the loss of *Men1*, as a hallmark of osteoblast aging.

## 3 DISCUSSION

We found that osteoblast‐specific *Men1* KO resulted in significant porosity, thinning of the cortical bone, decreased cortical mineral density, widening of the bone marrow cavity, and decreased mechanical strength. In addition, the bones of *Men1*‐deficient mice exhibited a decrease in osteoblast number and an increase in osteoclast number, resulting in a low BV and bone thinning. In vitro assays revealed that *Men1* deletion activated cellular senescence, and mTORC1 in osteoblasts through suppression of AMPK activity, which was rescued by metformin. Metformin suppressed the activation of mTORC1 through AMPK due to *Men1* deficiency and restored the low BV due to senescent osteoblasts in *Men1‐*deficient mice.

The present study thus revealed that loss of *Men1* is a hallmark of cellular senescence‐associated bone aging. As senescent cells accumulate in aged individuals, bone aging is clinically associated with osteoporosis (Farr et al., 2017). Osteoporosis is caused by aging, menopause, and lack of activity in daily life (Ukon et al., 2019). Several mouse models have been established for osteoporosis caused by menopause (Farr et al., 2019) or inactivity in daily life (Colaianni et al., 2017), whereas age‐related osteoporosis has generally been investigated using naturally or genetically aged mice (Silva et al., 2005). Since aged mice have also undergone menopause and exhibit reduced activity, aged mice do not represent a suitable model for specifically examining age‐related osteoporosis. We identified the loss of *Men1* as a single factor in the pathogenesis of age‐related osteoporosis in terms of cellular senescence. Therefore, this animal model can be used to obtain further insights into bone aging by cellular senescence through the measurement of the degree of age‐related osteoporosis and for the discovery of new drugs.

This study characterized osteoblast senescence both in vitro and in vivo. Senescent osteoblasts exhibited an increase in the mRNA levels of SASP factors, including *IL1α*, *IL6*, *IL8*, and *MMP3*, which were associated with an increase in osteoclast numbers. Consistent with our results, senescent cells around the bone tissue secrete SASP and lead to an increase in osteoclast numbers (Farr et al., 2017). Senescent osteoblasts can result in the fragility of cortical bones in the trunk. During bone formation, osteoblast senescence can lead to a decrease in BV and thickness of new bones, and metformin can restore the decreased BV. Osteoporosis is characterized by the deterioration of cortical and trabecular microstructures, bone fragility, and decreased osteogenic capacity (Ukon et al., 2019). Therefore, our model of osteoblast senescence‐associated bone aging in mice recapitulates some of these characteristics in the elderly human population. A previous report demonstrated that the removal of all senescent cells, including senescent osteoblasts, could alleviate osteoporotic phenotypes such as those pertaining to bone thickness, number of osteoclasts, number of osteoblasts, and MAR (Farr et al., 2017), which were the same factors found to be exacerbated by osteoblast senescence‐associated bone aging in the present study. Although various senolytic drugs are expected to be developed in the future, the pathophysiology and phenotypes revealed in this study can serve as biomarkers for evaluating the efficacy of drugs targeting cellular senescence‐associated bone aging.

We focused on *Men1* as a regulator of osteoblast senescence. Permanent *Men1* deficiency in bone from embryonic period reduces bone mass (Kanazawa et al., 2015; Liu et al., 2017). However, the relationship between *Men1* and cellular senescence in adults could not be evaluated because cellular senescence is deeply involved during the embryonic period, thus developmental *Men1* effect cannot be eliminated (Muñoz‐Espín et al., 2013). In this study, by using inducible conditional knockout mice, *Men1* deficiency in osteoblasts caused cellular senescence, which was associated with mTORC1 activation and induced bone aging. Consistent with our current results on the role of *Men1* in osteoblasts, several reports have shown that *Men1* deficiency in other cell types causes age‐related phenotypes such as impaired lymphocyte function and the development of dementia (Kuwahara et al., 2014; Leng et al., 2018; Suzuki et al., 2018). Loss of *Men1* in T cells promotes cellular senescence through mTORC1 activation (Suzuki et al., 2018). *MEN1*, a tumor suppressor gene, causes MEN1, an autosomal dominant disorder and hereditary tumor syndrome (Thakker et al., 2012). Patients with MEN1 develop osteoporosis early in life, regardless of tumor development (Marini et al., 2022) and tend to develop benign tumors, in contrast to patients with other hereditary tumor syndromes, who tend to develop malignant tumors (Thakker et al., 2012). Indeed, permanent *Men1* deficiency in early osteoblasts develop ossifying fibroma, a benign bone tumor in mice (Lee et al., 2018). Senescent cells are identified at high frequencies in benign tumors, whereas they are rarely detected in malignant tumors (Childs et al., 2014; Collado & Serrano, 2010). These clinical features of MEN1 support our results on cellular senescence in *Men1*‐deficient mice in vitro and in vivo. In a process known as oncogene‐induced senescence (OIS), cells become senescent in response to the activation of oncogenes, which promotes cancer development (Ou et al., 2021). The concept of OIS, induced by oncogenes, resembles that of cellular senescence by *Men1* gene deficiency. Although the overexpression of oncogenes immediately induces cellular senescence in vitro (Liu et al., 2018) and forms malignant tumors in vivo (Won & Choi, 2022), the loss of *Men1* appears to gradually accelerate cellular senescence, presumably through mTORC1 activation. Thus, an aging animal model with loss of *Men1* is consistent with the slow progression of natural aging. Our results suggest that *Men1* is deeply involved in cellular senescence‐associated aging and provide insights into new disease concepts.

This study had several limitations. First, the mechanism by which *Men1* deficiency leads to the bone‐aging phenotype has not yet been clearly elucidated. *Men1* is reported to be epigenetically involved in cell cycle progression, apoptosis, and the DNA damage response, which are important factors for cellular senescence (Kaji, 2012). Moreover, the BMP and transforming growth factor‐beta (TGF‐β) pathways, acting as major contributors to bone metabolism, have been associated with *Men1* (Kaji, 2012). However, the effect of *Men1* on these pathways depends on the cell type and does not necessarily facilitate bone formation (Troka et al., 2022). Because the BMP and TGF‐β pathways contribute to cellular senescence (Hayashi et al., 2016; Wei & Ji, 2018), loss of *Men1* may alter BMP or TGF/β signaling to induce cellular senescence. Furthermore, a direct involvement of *Men1* deficiency in age‐related osteoporosis has not been demonstrated. A previous study using mice with permanent *Men1* deficiency has demonstrated the absence of significant differences in bone‐related parameters in older age beyond 1 year (Liu et al., 2017), suggesting that inducible *Men1* deficiency, even in aged mice, does not exacerbate osteoporosis. In this study, expression of *Men1* was observed to be reduced with aging, suggesting the model used mimics age‐related osteoporosis. Second, assessing the degree of cellular senescence in the femur is challenging owing to the limited number of senescent osteoblasts. To solve this problem, we used a BMP‐induced bone formation model for a detailed assessment of cellular senescence in vivo, which allowed us to assess the degree of cellular senescence, as BMP accelerates mTORC1 and regulates cellular senescence (Kaneda et al., 2011; Karner et al., 2017). Third, although 6‐month‐old mice are typically used for senescence research, in this study, we used 2‐month‐old mice as young mice in accordance with the experiments on TAM‐induced *Men1* KO mice. Moreover, we selected 2‐month‐old mice based on the need for high knock‐out efficiency in inducible mice, following the protocol from a previous report (Zhong et al., 2015). However, the aging‐related results obtained in this study (Figure 1a, Figure S1) were similar to those of previous studies using 6‐month‐old mice as controls (Farr et al., 2016; Shim et al., 2022).

Nevertheless, in conclusion, we demonstrated that the loss of *Men1* is a critical driver of osteoblast senescence and established the first animal model of age‐related osteoporosis by osteoblast‐specific inducible *Men1* KO. This model could facilitate the study of the novel concept of cellular senescence‐associated osteoporosis in the context of clinical practice.

## 4 MATERIALS AND METHODS

### 4.1 Animals

Two‐month‐old and 24‐month‐old C57BL/6J mice were obtained from Charles River Laboratories Japan, Inc. (Kanagawa, Japan). *Men1* ^flox/flox^ mice were kindly provided by Dr. Masakatsu Yamashita (Ehime University). *Col1a1‐cre/ERT2* mice were kindly provided by Dr. Masaru Ishii (Osaka University, Osaka, Japan), *Col1a1‐GFP* and Ai9 (RCL‐tdT) mice were kindly provided by Dr. Satoru Otsuru (University of Maryland, College Park, MD, USA). Male mice were used for each experiment. *Men1* ^flox/flox^ mice were crossed with *Col1a1‐cre/ERT2* mice to generate mice harboring TAM‐induced homozygous deletion of *Men1* in osteoblasts. *Men1* ^flox/flox^ mice were used as controls for *Men1* ^flox/flox^; *Col1a1‐cre/ERT2* mice, both of which received TAM treatment. Immediately after weaning, age‐matched mice were randomly co‐housed independent of genotype. These mice were maintained on a normal chow diet with a 12‐h light/dark cycle. The following primer sets were used for genotyping by PCR to confirm model establishment: 5′‐GTGGTCAGGAGAGCAAATGAGTGT‐3′ and 5′‐CAGATCCCTCTGGCTATTCAATGG‐3′ for *Men1* wild‐type; 5′‐GCCATTTCATTACCTCTTTCTCCG‐3′ and 5′‐TACCACTGCAAAGGCCACGC‐3′ for *Men1* floxed allele; 5′‐CCCACATCCAGTCCCTCTTCAGCT‐3′ and 5′‐CATAAAATCGCAGCAGGTGGGCAA‐3′ for *Men1*‐deleted allele; 5′‐AGGTTCGTTCACTCATGGA‐3′ and 5′‐TCGACCAGTTTAGTTACCC‐3′ for *Cre*; 5′‐TGAACCGCATCGAGCTGAAGGG‐3′ and 5′‐TCCAGCAGGACCATGTGATCGC‐3′ for *Col1a1‐GFP*; and 5′‐GGCATTAAAGCAGCGTATCC‐3′ and 5′‐ and CTGTTCCTGTACGGCATGG‐3′ for *Ai9* (RCL‐tdT).

### 4.2 Micro‐CT analysis

A high‐resolution μCT scan (Skyscan 1272, Bruker, Billerica, MA, USA) was used at a resolution of 10 μm per voxel. Parameters, including BV, tissue volume, thickness, cortical porosity, mineral density, and bone marrow area, were analyzed using CTAn software (Bruker). Mineral density calibration was performed using a bone calibration phantom. Three‐dimensional reconstructed images were obtained using CTvox software (Bruker). The analysis area of the femur was set at 1.5 mm from the distal growth plate to 1.5 mm proximally.

### 4.3 RT‐qPCR

Total RNA was isolated using the TRIzol reagent (Thermo Fisher Scientific, Waltham, MA, USA). Bone preprocessing for in vivo assays was performed as previously described (Farr et al., 2016). Spine bone were used to assess general bone aging as reported (Farr et al., 2016). Briefly, after the bones were minced into small pieces, they were subjected to two sequential 30‐min collagenase digestions (Worthington Biochemical, Worthington, OH, USA). The total RNA was extracted from the remaining chips. Abundant osteoblasts in the remaining bone chips were confirmed according to GFP‐positive signals in the preprocessed bone chips of *Col1a1‐GFP* mice (Figure S3d). Complementary DNA was synthesized by ReverTra Ace (TOYOBO, Osaka, Japan) and then used as a template in qPCR with FAST SYBR Green Master Mix (Thermo Fisher Scientific). Target mRNA levels were normalized to reference gene expression. The median threshold cycle was compared with that of the control sample, and the fold difference between the reference and target genes was calculated. The internal controls were *Gapdh* for in vitro assays (osteoblasts) and *Hprt* for in vivo assays (osteoblast lineage‐enriched bone cells). The primer sequences used in RT‐qPCR are listed in Table S1.

### 4.4 *Cre/loxP* recombination by TAM treatment

The dose and schedule of TAM administration (10 mg/kg/day for 4 days) and age of mice (4 weeks old) were determined according to a previous report, in which the authors confirmed the minimum TAM dosage for reliable *Cre/loxP* recombination with little effect on the bone formation ratio (Zhong et al., 2015). The TAM solution (20 mg/mL) was prepared by dissolving TAM powder (Sigma‐Aldrich, St. Louis, MO, USA) in ethanol and diluted 20‐fold with corn oil (C8267, Sigma‐Aldrich). For femur analysis, *Men1* ^flox/flox^ (control) and *Men1* ^flox/flox^; *Col1a1‐cre/ERT2* (*Men1* KO) mice were treated with 10 mg/kg TAM per day for 4 days at 4, 6, and 8 weeks of age. The mice were euthanized and analyzed at 9 weeks of age. *Men1* deletion by *Cre/loxP* recombination was confirmed by PCR of calvaria bone (Figure S3c). For BMP‐induced ectopic bone analysis, control and *Men1* KO mice underwent BMP collagen sponge implantation at 4 weeks of age. Osteoblasts in the BMP‐induced ectopic bone were visible 10 days after implantation (Hashimoto et al., 2020). To delete the osteoblast *Men1* gene at the appropriate time, mice were treated with TAM (10 mg/kg/day for 4 days) at 8–11 and 15–18 days after the surgery and were euthanized 21 days after the surgery. *Men1* deletion by *Cre/loxP* recombination was confirmed by PCR of calvaria bone (Figure S3c). The efficiency of *Cre/loxP* recombination was confirmed in *Col1a1‐cre/ERT2*; *Col1a1‐GFP*; Ai9 mice (Figure S3b).

### 4.5 Biomechanical testing

Three‐point bending tests were performed using an MZ‐500 system (Marutoh, Mie, Japan) to investigate the biomechanical strength of the femur bone. The bones were placed on a pedestal, and force was applied to the center of the diaphysis at a rate of 2 mm/s until failure (ultimate load) (Figure 1g). The ultimate load (N), displacement at fracture (mm), stiffness (N/mm), and post‐yield displacement (mm) were determined from a load–displacement diagram.

### 4.6 Histomorphometric analysis

Double labeling by subcutaneous injection of tetracycline (20 mg/kg) and calcein (10 mg/kg) was performed 5 and 2 days before euthanasia, respectively. The resected samples were fixed in 70% ethanol, stained with Villanueva, and embedded in methacrylic acid (Wako Pure Chemical Industries, Kanagawa, Japan). Quantitative data from double‐labeled tissue sections were generated based on measured values using Histometry RT Digitiser (System Supply, Nagano, Japan) as a bone morphometry system and CSS‐840 Trabecular Bone Measurement Version as software (System Supply). The following histomorphometric parameters were measured: MAR, BFR/BS, BV/TV, trabecular thickness (Tb.Th), number of osteoblasts per bone surface (N.Ob/BS), osteoblast surface per bone surface (Ob.S/BS), number of osteoclasts per bone surface (N.Oc/BS), and osteoclast surface per bone surface (Oc.S/BS).

### 4.7 Osteoblast isolation from the neonatal murine calvaria

Primary osteoblasts were obtained from the calvaria of 2–3‐day‐old neonatal *Men1* ^flox/flox^ mice as previously described (Yoshida et al., 2022). Briefly, calvaria were minced and digested in alpha‐minimal essential medium (Nacali Tesque, Kyoto, Japan), 0.176% NaHCO_3_, and 0.008% collagenase type II (Worthington Biochemical) at 37°C for 15 min seven times. Supernatants from the third to seventh digestions were collected. After centrifugation, cell suspensions were incubated in 10‐cm dishes with alpha‐minimal essential medium (α‐MEM) (Nacali), 10% fetal bovine serum, and 1% penicillin–streptomycin at 37°C with 20% O_2_ and 5% CO_2_. After 1 week, confluent cells (passage 0) were used for experiments.

### 4.8 Adenovirus infection of osteoblasts

Osteoblasts from *Men1* ^flox/flox^ mice were passaged into 24‐well plates at a density of 5 × 10^5^ cells per 75 cm^2^ (passage 1) and infected with eGFP adenovirus (Ad‐GFP, [Cat. No: 1060, Vector Laboratories, Malvern, PA, USA]: Control) or Cre recombinase adenovirus (Ad‐Cre‐GFP [Cat. No: 1700, Vector Laboratories]; *Men1* KO) at a multiplicity of infection of 100 for 48 h.

### 4.9 Replicative senescence by long‐term culture

One week after adenovirus infection, cells were passaged in a 10‐cm dish at 5 × 10^5^ cells per 75 cm^2^ (passage 2) and then passaged to a 10‐cm dish at the same density every other week. Passage‐3 and passage‐6 cells were used for the experiments as the low replicative stress and high replicative stress group, respectively, since a previous report suggested that cellular senescence starts after passage 6 under normal conditions (Parrinello et al., 2003). SA‐β‐Gal staining and RT‐qPCR were performed using passage‐3 and passage‐6 cells. Western blotting was performed on passage‐3 cells with or without 10 mM metformin treatment for 48 h.

### 4.10 SA β‐gal staining

SA β‐Gal staining was performed using a Senescence β‐galactosidase Staining Kit (#9860, Cell Signaling Technology, Danvers, MA, USA). Green‐stained cells were counted in 10 random fields under a microscope (BZ‐X800, Keyence, Osaka, Japan) with a 10× magnification objective lens and calculated as the percentage of positive cells. To avoid non‐specific staining due to cell confluence, the assay was performed using subconfluent cells.

### 4.11 Immunoblotting

After the cells were lysed with RIPA buffer (Nacalai Tesque), the protein concentrations were determined using a BCA assay (Thermo Fisher Scientific). The lysates were mixed with 5× sodium dodecyl sulfate sample buffer and boiled for 5 min. Equal amounts of protein were separated on 4–12% Bis‐Tris gels (Life Technologies, Carlsbad, CA, USA) and transferred onto polyvinylidene difluoride membranes (Nippon Genetics, Tokyo, Japan). Membranes were blocked with Tris‐buffered saline containing 5% skim milk and Tween 20 at room temperature, incubated with the primary antibody in Can Go Signal Solution 1 (Toyobo) at 4°C overnight, and then incubated with the secondary antibody in Can Go Signal Solution 2 (Toyobo) for 1 h at room temperature. The ECL Plus Western Blotting Detection System kit (Thermo Fisher Scientific) was used to detect immunoreactive proteins. The following antibodies were used for western blotting at the indicated dilutions: rabbit monoclonal anti‐4E‐BP1 (#9644, 1:1000, Cell Signaling Technology), rabbit monoclonal anti‐phospho‐4E‐BP1 (#2855, 1:1000, Cell Signaling Technology), rabbit monoclonal anti‐p70 S6 kinase (#9202, 1:1000, Cell Signaling Technology), rabbit monoclonal anti‐phospho‐p70 S6 kinase (#9234, 1:1000, Cell Signaling Technology), rabbit polyclonal anti‐AMPKα (#2532, 1:1000, Cell Signaling Technology), rabbit monoclonal anti‐phospho‐AMPKα (#2535, 1:1000, Cell Signaling Technology), rabbit monoclonal anti‐Akt (#24691, 1:1000, Cell Signaling Technology), (#2532, 1:1000, Cell Signaling Technology), rabbit monoclonal anti‐ Phospho‐Akt (Ser473) (#4060, 1:1000, Cell Signaling Technology), rabbit monoclonal anti‐p44/42 MAPK (Erk1/2) (#4695, 1:1000, Cell Signaling Technology), rabbit monoclonal anti‐Phospho‐p44/42 MAPK (Erk1/2) (Thr202/Tyr204) (#4370, 1:1000, Cell Signaling Technology), rabbit monoclonal anti‐GSK‐3β (#12456, 1:1000, Cell Signaling Technology), rabbit monoclonal anti‐Phospho‐GSK‐3β (Ser9) (#9323, 1:1000, Cell Signaling Technology), rabbit polyclonal anti‐GAPDH (#2118, 1:1000, Cell Signaling Technology), and horseradish peroxidase‐conjugated goat anti‐rabbit IgG (#7074, 1:1000, Cell Signaling Technology). Band intensity was quantified using the ImageJ software (NIH, MD, USA).

### 4.12 BMP‐induced ectopic bone model

A collagen sponge (Colla Cote, Zimmet Dental, CA, USA) was cut into cylindrical shape (diameter of 5 mm and height of 2 mm) for establishing the BMP‐induced ectopic bone model. Recombinant human BMP‐2 (Osteopharma Inc., Osaka, Japan) derived from E.coli was used. Recombinant human BMP‐2 (1.5 μg) was soaked into the cylindrical collagen sponge. The sponge was then freeze‐dried (BMP pellets). Under anesthesia, the BMP pellet was implanted underneath the dorsal fascia of mice. Metformin was administered orally in the drinking water at a concentration of 300 μg/mL starting 2 weeks after the surgery. The dose of metformin was set based on the effective dose for extending lifespan (Anisimov et al., 2011).

### 4.13 Histological analysis

The excised samples were fixed in 10% neutral‐buffered formalin, decalcified by ethylenediaminetetraacetic acid, embedded in paraffin wax, and cut at 3 μm thickness. Hematoxylin and eosin staining was performed according to standard protocols. Paraffin‐embedded sections were deparaffinized and dehydrated for immunostaining. The antigens were activated in 10 mM citrate buffer at 95°C for 10 min. After quenching endogenous peroxidase activity with methanol containing 3% H_2_O_2_ for 10 min, the sections were blocked with Blocking One (Nacalai Tesque) for 1 h at room temperature. Sections were then incubated with a primary antibody overnight at 4°C, followed by incubation with a horseradish peroxidase‐labeled secondary antibody for 1 h. Finally, the labeled sections were stained with Histofine Simple Stain Mouse MAX PO (Nichirei Bioscience, Tokyo, Japan) and counterstained with hematoxylin. Rabbit monoclonal anti‐CDKN2A/p16INK4a (ab211542, 1:250; Abcam, Cambridge, UK), rabbit monoclonal anti‐ Phospho‐S6 Ribosomal Protein (Ser240/244) (#5364, 1:500, Cell Signaling Technology), rabbit monoclonal anti‐Phospho‐4EBP1 (Thr37/46) (#2855, 1:800, Cell Signaling Technology), rabbit polyclonal anti‐RANKL (bs‐0747R, 1:200, Bioss Inc, Massachusetts, USA), rabbit polyclonal anti‐OPG (ab216484, 1:100, Abcam), and rabbit monoclonal anti‐phospho‐AMPKα (Thr172) (#2535, 1:50, CST) were used as the primary antibody. The number of CDKN2A/p16INK4a‐positive cells on the entire perimeter of the BMP‐induced ectopic bone was counted manually. Frozen sections, which were 5 μm thick, were utilized to identify naturally occurring fluorescent proteins like tdTomato and GFP or SA β‐gal. For fluorescent protein, these sections were nuclear stained with 4′,6‐diamidino‐2‐phenylindole solution (Dojindo Laboratories, Kumamoto, Japan) and mounted with Prolong Diamond Antifade Mountant (Thermo Fisher Scientific). BZ‐X800 (Keyence) was used to observe and capture the fluorescence images. The SA β‐gal staining was performed using Senescence β‐galactosidase Staining Kit (#9860, Cell Signaling Technology).

### 4.14 Statistics

Statistical analysis was performed using GraphPad Prism version 8.4.1 for Windows (GraphPad Software, San Diego, CA, USA) with two‐tailed Student's *t*‐test, one‐way ANOVA followed by.

Sidak's test or Fisher's LSD test. Values are presented as the mean ± standard deviation. Statistical significance was set at *p* < 0.05.

## AUTHOR CONTRIBUTIONS

Y. U.: Conceptualization, methodology, software, formal analysis, investigation, writing—original draft, funding acquisition. T. K.: Conceptualization, validation, resources, writing—review and editing, supervision, project administration, funding acquisition. H. H.: Investigation, resources, writing—review and editing. T. K.: Investigation, resources, writing—review and editing. M. B.: Investigation, resources, writing—review and editing. J. K.: Writing—review and editing. D. T.: Writing—review and editing. S. N.: Resources, writing—review and editing. M. I.: Writing—review and editing. T. F.: Writing—review and editing. Y. K.: Writing—review and editing. T. F.: Writing—review and editing. S. T.: Writing—review and editing. T. Y.: Writing—review and editing, supervision. S. O.: Writing—review and editing, supervision. S. O.: Writing—review and editing, supervision. M. Y.: Conceptualization, resources, writing—review and editing, supervision, project administration. T. I.: Conceptualization, resources, writing—review and editing, supervision, project administration.

## FUNDING INFORMATION

This study was supported by JSPS KAKENHI Grant Numbers 20K09479, 22K20960 and 23K15689, the Asahi Kasei Joint Research Foundation, and the Nakatomi Foundation.

## CONFLICT OF INTEREST STATEMENT

The authors declare that they have no conflict of interest.

## STUDY APPROVAL

All experiments were approved by the Institutional Animal Committee of Osaka University.

## Supporting information


Figure S1.

Figure S2.

Figure S3.

Figure S4.

Table S1.

## Data Availability

The datasets generated and analyzed in the current study are available from the corresponding author upon reasonable request.
