# Supplementary figures and images for: Cellular senescence by loss of Men1 in osteoblasts is critical for age‐related osteoporosis
Source: Aging Cell. 2024 Jun 22;23(10):e14254. doi: 10.1111/acel.14254 (PMC11464108; doi:10.1111/acel.14254)

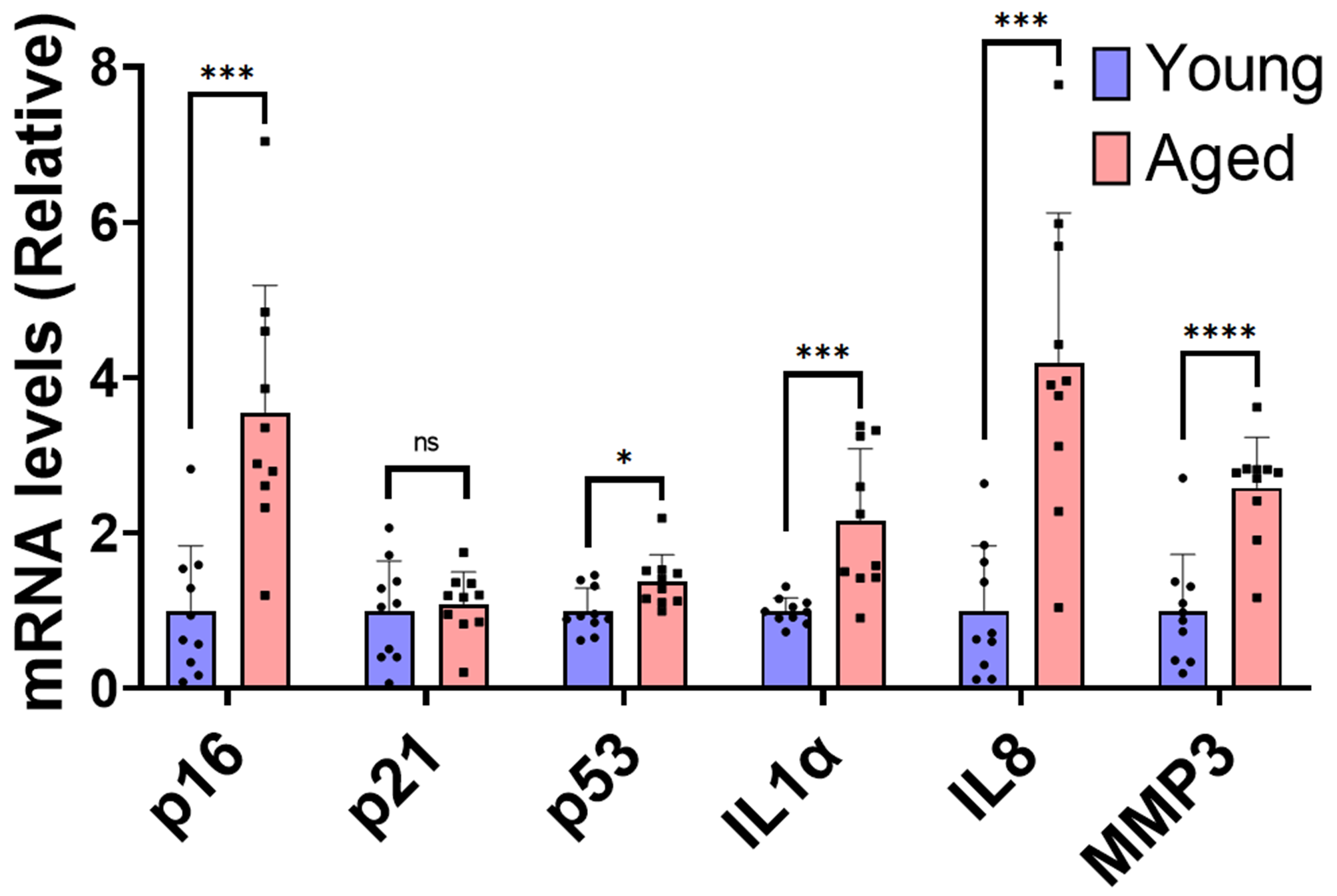

Supplement: Supplementary file 1 — Figure S1. [file ACEL-23-e14254-s004.tif]

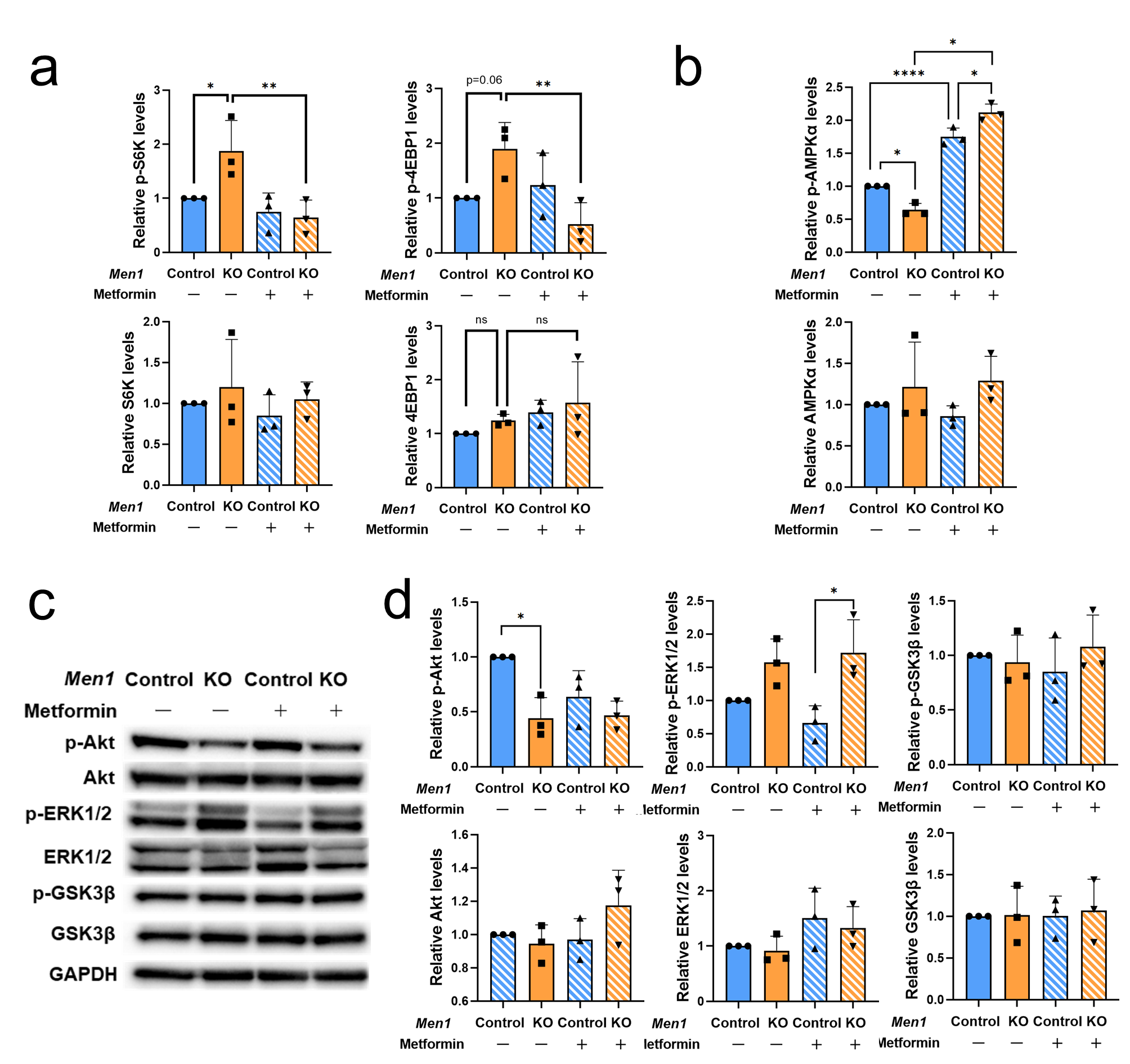

Supplement: Supplementary file 2 — Figure S2. [file ACEL-23-e14254-s003.tif]

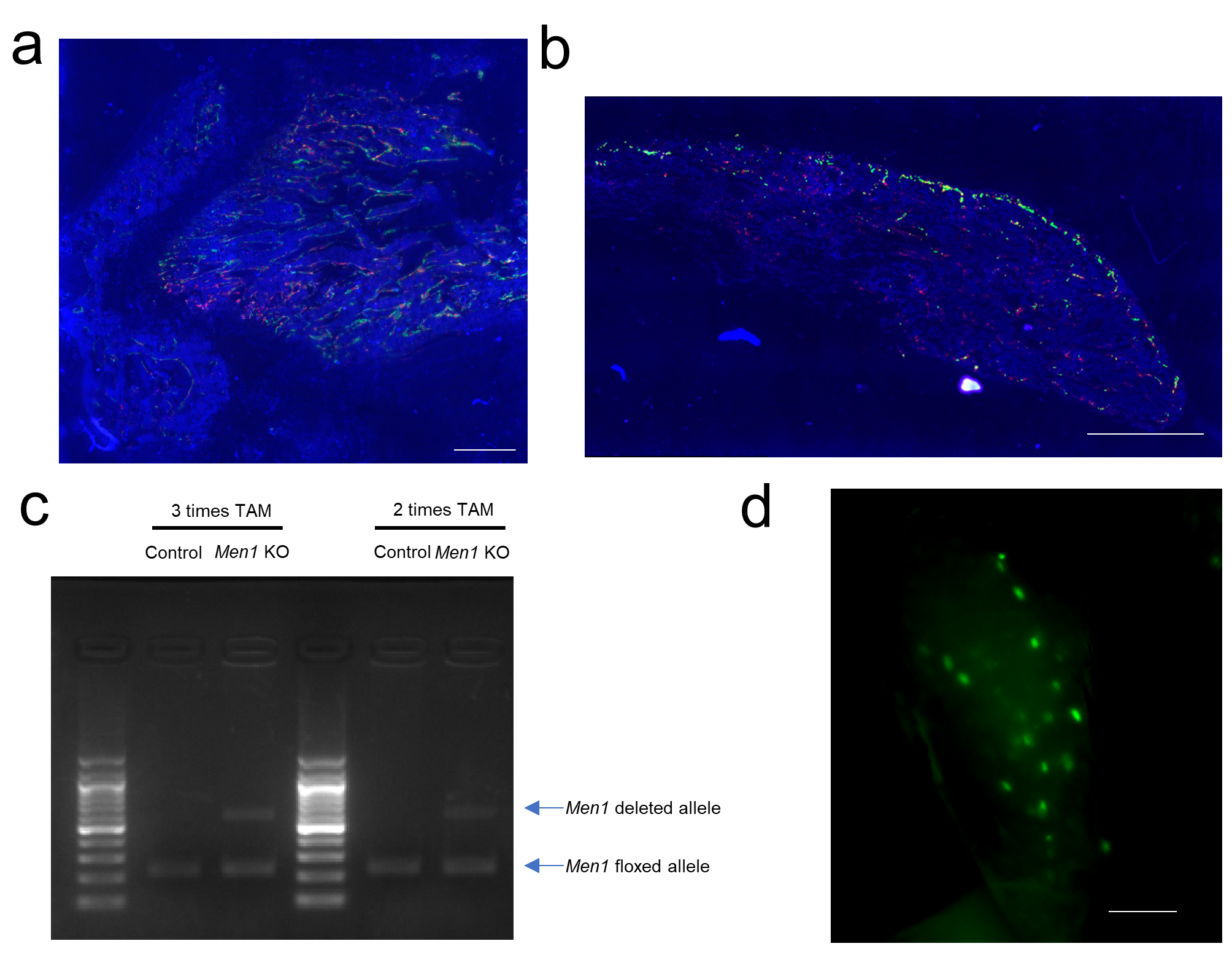

Supplement: Supplementary file 3 — Figure S3. [file ACEL-23-e14254-s002.tif]

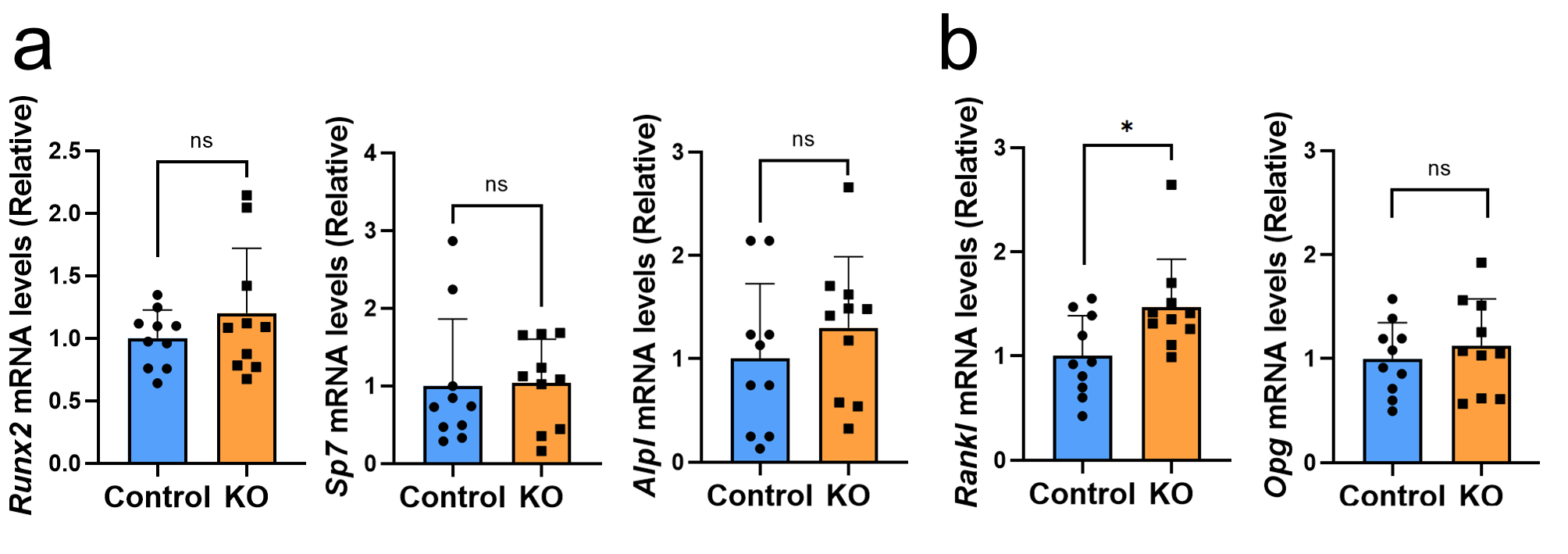

Supplement: Supplementary file 4 — Figure S4. [file ACEL-23-e14254-s005.tif]
