# Supplementary material for: Cellular senescence by loss of Men1 in osteoblasts is critical for age‐related osteoporosis
Source: Aging Cell. 2024 Jun 22;23(10):e14254. doi: 10.1111/acel.14254 (PMC11464108; doi:10.1111/acel.14254)
Supplement: Supplementary file 5 — Table S1. [file ACEL-23-e14254-s001.docx]

| Gene | Forward | Reverse |
| --- | --- | --- |
| Men1 | TCATCCCCAACCTGCTGAAG | GCATTCTGGGTCCTGGAGAG |
| p16 | TGGAACTTCGCGGCCAATC | CGCTAGCATCGCTAGAAGTG |
| p21 | GGAGGAGCATGAATGGAGACAGA | TCGGACATCACCAGGATTGGA |
| p53 | GGACCCTGGCACCTACAATG | GGAAGGAAAGTAGGCCCTGG |
| IL1a | TGCAAGCTATGGCTCACTTC | GATACTGTCACCCGGCTCTC |
| IL6 | AGCCAGAGTCCTTCAGAG | CCACTCCTTCTGTGACTC |
| IL8 | TTGGAGCCAAGGCAAGAACAC | GGAGAGGCATCCGGTTCACA |
| MMP3 | AGTGGATCTTCGCAGTTGGA | CACAGGATGCCTTCCTTGGAT |
| Rankl | TCCTGAGACTCCATGAAAACGCAG | GCCACATCCAACCATGAGCCTTC |
| Opg | TGAGAGAACGAGAAAGACCTGC | CGGATTGAACCTGATTCCCTAT |
| Runx2 | AGGCTGTGCACCAAGTAGAC | TGCTTCAAGCTACCACACACA |
| Sp7 | AGGCACAAAGAAGCCATAC | AATGAGTGAGGGAAGGGT |
| Alpl | CCAGCAGGTTTCTCTCTTGG | CTGGGAGTCTCATCCTGAGC |
| Gapdh | GGGTGTGAACCACGAGAAAT | ACTGTGGTCATGAGCCCTTC |
| Hprt | AGATGAGCGCAAGTTGAATCTG | GATGGCCACAGGACTAGAACA |
